# Application of a novel rectangular filtering microfluidic device for microfilarial detection

**DOI:** 10.3389/fvets.2022.1048131

**Published:** 2023-01-06

**Authors:** Sariya Asawakarn, Alongkorn Pimpin, Wutthinan Jeamsaksiri, Witsaroot Sripumkhai, Wanarit Jitsamai, Piyanan Taweethavonsawat, Prapruddee Piyaviriyakul

**Affiliations:** ^1^Biochemistry Unit, Department of Veterinary Physiology, Faculty of Veterinary Science, Chulalongkorn University, Bangkok, Thailand; ^2^Biomarkers in Animal Parasitology Research Group, Chulalongkorn University, Bangkok, Thailand; ^3^Department of Mechanical Engineering, Faculty of Engineering, Chulalongkorn University, Bangkok, Thailand; ^4^Micro/Nano Electromechanical Integrated Device Research Unit, Faculty of Engineering, Chulalongkorn University, Bangkok, Thailand; ^5^Thai Microelectronics Center (TMEC), Chachoengsao, Thailand; ^6^Parasitology Unit, Department of Veterinary Pathology, Faculty of Veterinary Science, Chulalongkorn University, Bangkok, Thailand

**Keywords:** detection, filter, microfilaria, microfluidic, rectangle

## Abstract

The rectangular filtering microfluidic chip was invented using microfluidics device fabrication technology and can separate living microfilariae from blood samples without a syringe pump. The diagnostic results are highly effective. The device is based on the principle of separating millions of blood cells from microfilariae using a rectangular filter structure. It disperses fluid evenly into the flow-passage channel, and its rectangular filter structure is the key to success in reducing the pressure and separating blood cells from microfilariae effectively. The flow rate and blood cell concentration were optimized in our study. The chip is intended to be a point-of-care device that can reduce the use of superfluous instrumentation in the field. The technology is designed to be rapid, accurate, and easy-to-use for all users, especially those in remote areas.

## 1. Introduction

Canine and feline filariasis are vector-borne parasitic infections of concern to public health in several countries including Thailand. Filariasis in dogs and cats is caused by several species of filarids, such as *Dirofilaria immitis, Dirofilaria repens, Brugia malayi, Brugia pahangi*, and *Acanthocheilonema reconditum*. Two of these species, *D. immitis* and *B. pahangi*, have been reported in Thailand (1, 2). Microfilariae can be detected by routine methods such as thick blood smear or fresh smear and modified Knott's test. Although the thick blood smear method is conventional, it has some limitations. For example, the method may result in false negatives at low densities of microfilariae (3). Therefore, the modified Knott's test, which concentrates microfilariae by centrifugation and marks specific species by Giemsa staining, was proposed. However, this technique comprises several steps and is time-consuming (4). Recently, the merits of microfluidic technologies have inspired many researchers to use them to develop new diagnostic methods. These technologies are widely applied in relation to nematodes, including for the diagnosis of diseases in humans, animals, and plants (5–7).

Past work used microfluidic techniques to detect microfilariae in human and feline blood samples by using filtered microfluidic devices and semi-automated devices (7, 8). However, these devices are not convenient for use by veterinarians in field settings and animal hospitals/clinics. Not only are additional instruments, such as syringe pumps, needed but the diagnostic protocol also includes several steps to remove unwanted contamination for clear visualization of the results. These requirements are mainly due to the inability to successfully physically separate microfilariae from other suspended objects, such as blood cells, specifically at the detection spot.

This study aimed to design a novel flow-through microfluidic device to isolate microfilariae from blood samples. The goal was to isolate microfilariae to the detection spot without other suspended objects obscuring vision, which would facilitate more accurate diagnosis, shorter preparation times, and ease of use compared to previous approaches. In addition, the device should be robust and suitable for use in a field setting for the detection, isolation, and subsequent culture for drug screening of microfilaria and/or genera of intracellular bacteria such as *Wolbachia* since the results of drug screening tests are necessary for veterinarians to plan for the treatment, prevention, and control of filariasis in animals.

## 2. Materials and methods

### 2.1. Microfluidic device design

**Figure 1:**
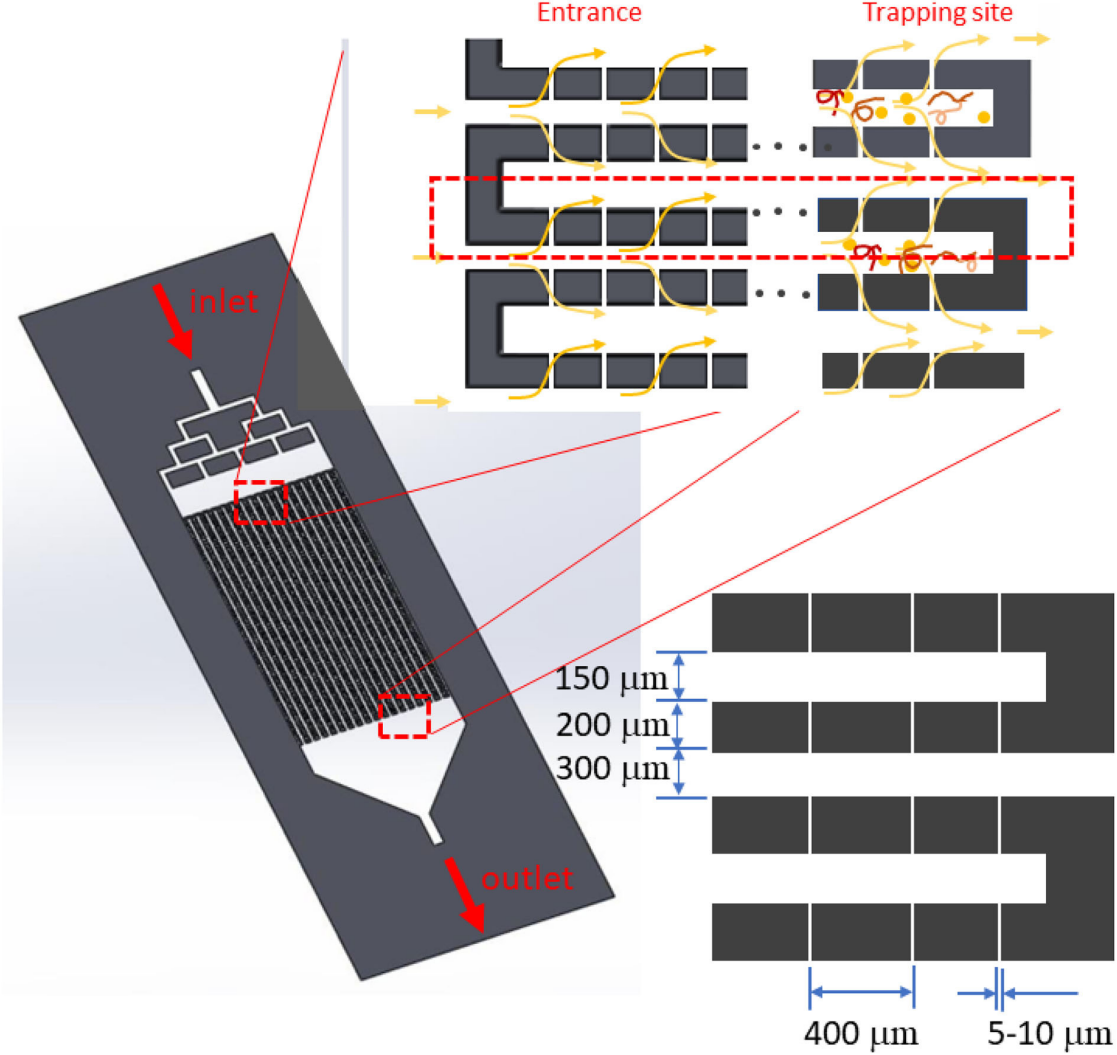
System design with long flow passages. Between the inlet and outlet passage, 5-μm micro-capillaries are placed on both the right- and left-hand walls. Microfilariae are captured at the end of each inlet flow passage.

The device was designed as a rectangular filtering structure consisting of 20 long flow-passages with small capillaries (about 100 openings) on the side walls along the channel length as shown in Figure 1. Small capillaries were located in the middle and divided the inlet and outlet flow passages. The passage width on the inlet side was 150 μm, and that on the outlet side was 300 μm. The passage length was around 25 mm. The capillaries were about 5 μm wide, with a length (equal to the wall thickness) of 200 μm. The height of the flow passage was adjustable and chosen to be 20–30 μm. The inlet manifold was designed to help distribute the blood sample evenly in each flow passage. Small suspended particles would be carried with the flow to the outlet, while larger ones would not. Large particles such as nematodes would be accumulated at the trapping site at the end of the inlet flow passage. Thus, the trapped particles would accumulate at a certain location, resulting in ease of microscopic investigation. Regarding fabrication, the rectangular filtering microfluidic device was built using a soft lithography process with a polydimethylsiloxane (PDMS) silicone material bonded on a glass slide (9).

### 2.2. Mathematical model

**Figure 2:**
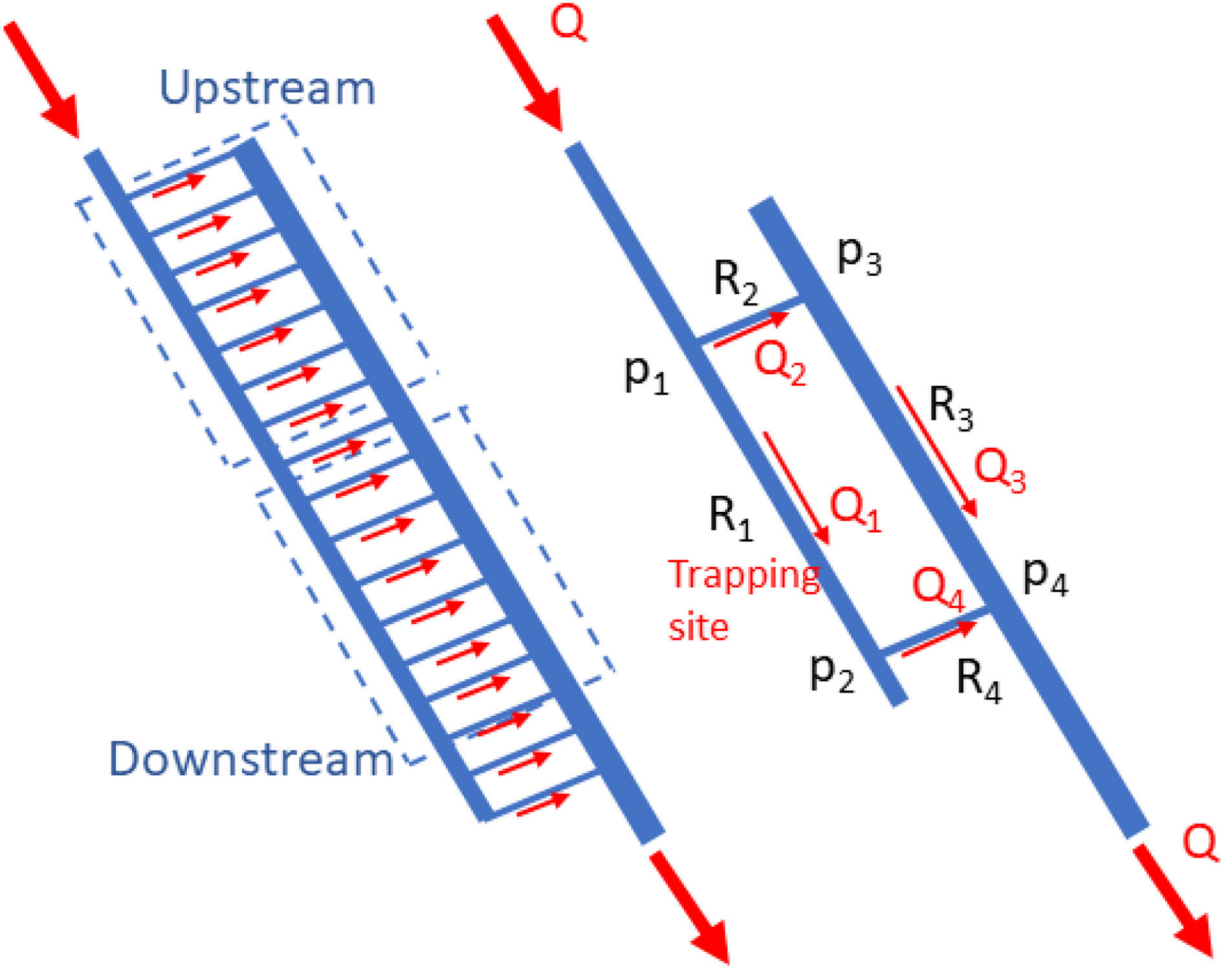
Simplified mathematical model for the two groups of micro-capillaries passing into the upstream and downstream micro-capillary.

The system could be modeled as shown in Figure 2. Capillaries connected between the inlet and outlet flow passages were divided into two groups that were modeled as an upstream and downstream capillary (consisting of small capillaries). With the model, the flow resistance (Pa.s^3^/m) at different locations (R_1_ [an inlet passage and trapping site], R_2_, R_3_, and R_4_ [an outlet passage]) could be calculated for a given total flow rate (10). The flow resistance is well known and can be expressed as Equation 1 (11),


(1) $$R=\frac{12\mu L}{wh^{3}\left(1-\frac{h}{w}\left(\frac{192}{\pi^{5}}\sum_{n=1,3,5}^{\infty }\frac{1}{n^{5}}\tan h\left(\frac{n\pi w}{2h}\right)\right)\right)},$$

where μ is viscosity (Pa.s), *L* is the channel length (m), *w* is the channel width (m), and *h* is the channel height (m).

Regarding the principle of mass conservation, the flow rates (m^3^/s) at each section could be calculated as Q_1_ = Q_4_, Q_2_ = Q_3_, and Q = Q_1_ + Q_2_, while the pressure drop (Pa) across the capillaries is P_1_ – P_4_ = Q_1_ (R_1_ + R_4_) = Q_2_ (R_2_ + R_3_). Using these equations at a given flow rate at the main inlet Q, the average flow rate in the flow passages (Q_1_, Q_2_, Q_3_, and Q_4_) and average pressure drop across the flow passages and the upstream and downstream capillaries (P_1_ – P_4_, P_1_ – P_3_, and P_2_ – P_4_) could be calculated, consecutively.

### 2.3. Microfluidic chip validation

The chip efficacy was first evaluated by inspecting the chip's durability, including checking for cracks and/or fluid leakage at different injecting flow rates. In addition, the dilution ratio of the blood sample was investigated by the visualization of blood clogging around the capillary that might occur when the flow rate and pressure drop are too high. Blood (1 mL of total volume) was diluted with normal saline solution to prepare samples with 1x, 2x, 5x, 10x, 20x, 50x, and 100x dilution ratios and injected into the chip at the inlet port. The flow rates were controlled by a syringe pump (Chemyx, USA) at 0.5, 1, and 1.8 mL/min. Images were captured under a light inverted microscope using the Motic Images Plus III software. Finally, the optimal flow rate and dilution ratio were validated by a veterinarian who injected the blood sample by hand.

### 2.4. Microfilariae detection and parasite identification

The efficiency of the chip was further examined to identify the lowest number of microfilariae that could be detected in the blood sample. Infected canine blood samples were collected from small animal hospitals and clinics in Bangkok and its vicinity. The research protocol was approved by the Chulalongkorn University Animal Committee (Approval No. 1731010). The samples were evaluated using the dilution technique at 20x and 50x, with an initial density of approximately 150 nematodes/mL. The initial density of microfilarial count was quantitated by three lines thick smear and Giemsa staining technique.

First, all canine blood samples were collected in blood collection tubes containing EDTA. Twenty microliters of the blood sample was diluted with normal saline solution. One milliliter of the diluted blood sample was then manually injected into the rectangular filtering microfluidic device in 1 min. Thereafter, the blood cells still accumulating in the system were flushed out using normal saline with the same flow rate (~1 mL/min). Finally, the microfilariae trapped inside the device were counted and observed under an inverted light microscope.

In a further investigation, the microfilariae were flushed out of the microfluidic chip by normal saline to the opposite direction and the species were identified by PCR (12). The procedure was as follows. Microfilariae from the chip were collected in 70% ethanol and subjected to DNA extraction according to the manufacturer's protocol (Qiagen, Germany). PCR was performed targeting the 5.8S-ITS2-28S regions using the primers DIDR-F1 (5'AGTGCGAATTGCAGACGCATTGAG'3) and DIDR-R1(5'AGCGGG-TAATCACGACTGAG TTGA'3) following a previously published method (8). The PCR reaction mix consisted of 10 μl of 2x ViRed Taq Master Mix (Vivantis, Malaysia), 0.5 μM of each primer, and 2 μl of DNA. The cycling conditions were as follows: 35 cycles of 94°C for 30 s, 60°C for 30 s, 72°C for 30 s, and 94°C for 2 min for pre-denaturation, with a final extension step at 72°C for 7 min. The PCR products were then subjected to gel electrophoresis, purified, and sequenced.

## 3. Results

### 3.1. Prototype and simulation results

**Figure 3:**
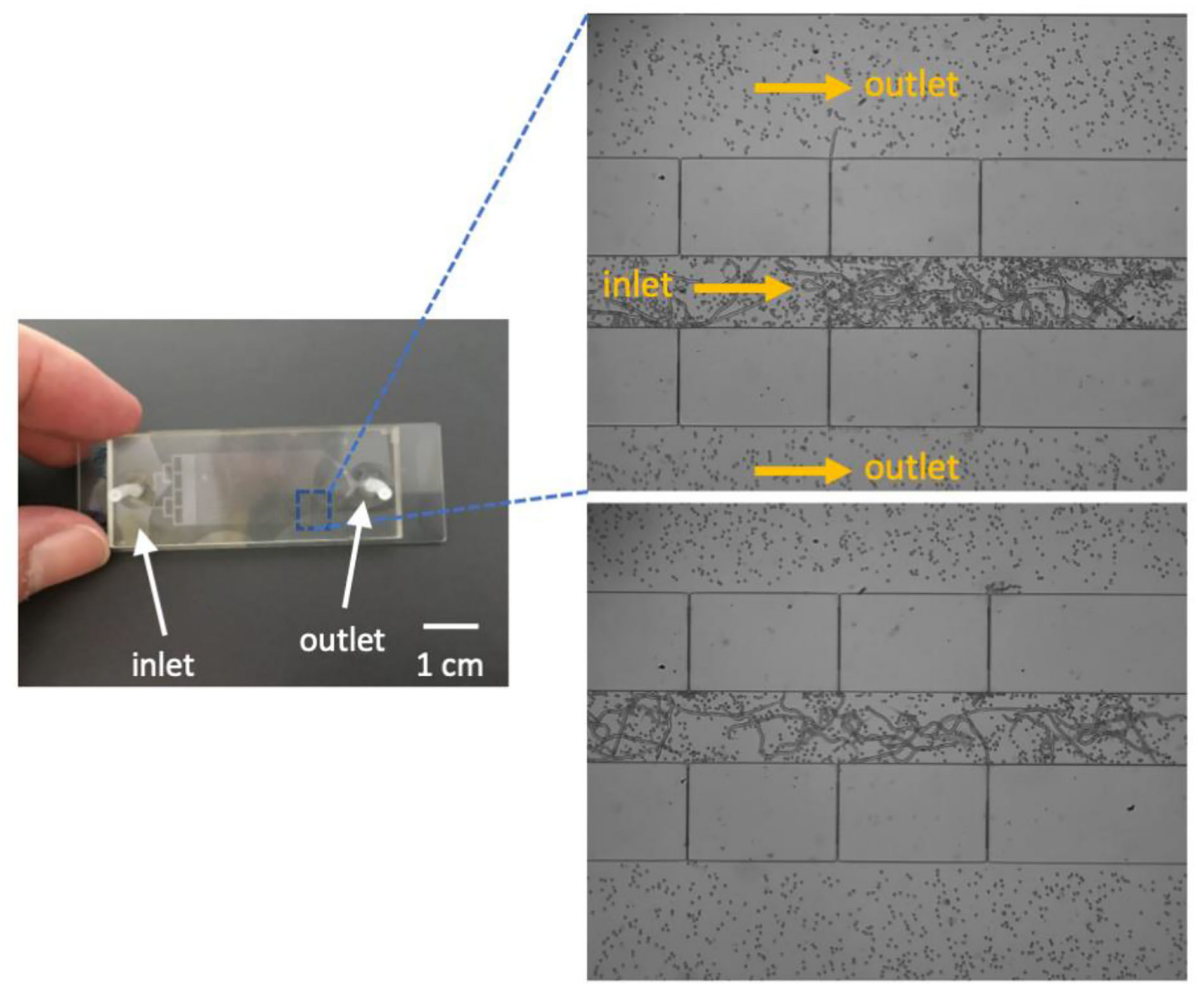
A prototype of the microfluidic chip **(left)** and trapped microfilariae **(right)**. Fluid flow direction is indicated by arrows in the image.

The PDMS rectangular filtering microfluidic chip is showed in Figure 3. The designed manifold at the inlet could disperse the sample evenly to the flow-passage channel. At an appropriate flow rate of 1 mL/min, no blood clogging was observed, and blood parasites were successfully trapped at the end of the flow passage as expected.

**Figure 4:**
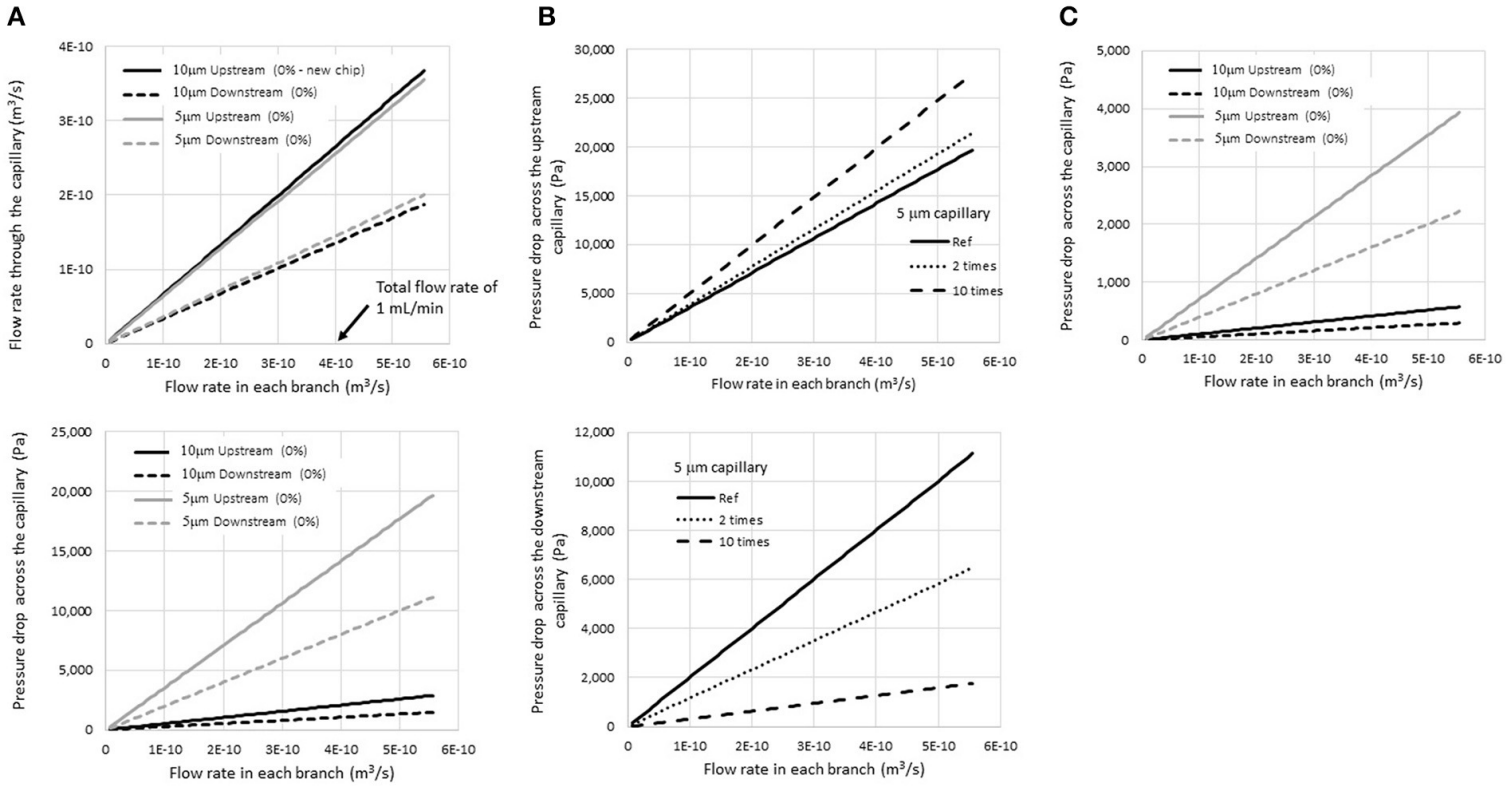
Simulation results of **(A)** the flow rate and pressure drop across the upstream and downstream capillary for a given total injecting flow rate for 5- and 10-μm-wide capillaries, **(B)** the pressure drop at the 5-μm upstream and downstream capillary when the flow resistance increase due to the accumulation of blood cells in the inlet flow passage. The calculation assumed a two-fold and ten-fold increase in flow resistance, and **(C)** the pressure drops when the viscosity was reduced five-fold.

To separate millions of blood cells from microfilaria, the flow rate and pressure drop in the microfluidic chip are very important parameters that define its utility and limitations. The results of the simulation suggested that when the total flow rate at the inlet was varied from 0 to 1 mL/min (or 4 × 10^−10^ m^3^/s), the flow rate and pressure drop across the upstream and downstream capillary increased as shown in Figure 4A. For the current design, where the inlet, outlet, and capillary widths are 150, 300, and 5 μm, respectively, the flow rate and pressure drop upstream were about two times higher than those downstream. Thus, the suspended particles tended to flow out of the filtering structures at the upstream location immediately as they passed through the system. This helped to rapidly eliminate small unwanted particles from the system. With a relatively short flow passage, unwanted particles would accumulate near the trapping site and reduce the visibility of targets for the microscopist. Thus, one key design principle involved increasing the length of the main flow passage as much as possible.

Regarding the capillaries, the pressure drops across the capillaries significantly increased when the capillary size was reduced from 10 to 5 μm, while the flow rate was not affected much. The average pressure drop across the upstream capillaries increased to 15 kPa when the sample was injected at a flow rate of 1 mL/min at the inlet. A high pressure drop would help eliminate unwanted particles efficiently; however, it may also damage the microchip structure. Thus, adjustment of the flow rate is vital to achieve high efficacy of the filtering device.

The situation in which some trapped particles started accumulating at the end of the flow passage was also studied. This condition caused an increase in the flow resistance R_1_, and the flow rate at the upstream and downstream capillary, as well as the pressure drop, would be altered accordingly.

In Figure 4B, the pressure drop at the upstream and downstream capillary is presented when the flow resistance at the inlet flow passage was increased two-fold and ten-fold compared to a new chip (labeled as “reference” in the graph). When particles were trapped, the pressure drop across the upstream flow passage increased, while that across the downstream passage decreased. Therefore, the possibility that unwanted particles would squeeze out through the upstream capillary increased. On the other hand, the leakage of trapped particles at the trapping site would be reduced after the experiment passes by.

Moreover, the device would be automatically protected from an overfull capacity by its design. According to the mathematical model, if the flow resistance increases significantly (due to the existence of trapped particles), the pressure drop at the upstream capillary would increase to a critical level at which all suspended particles would pass out from the filtering device immediately at the upstream location. A small number of particles would move deep into the flow passage and block the visibility of targets of interest at the end of the flow passage.

However, an increase in the pressure drops to a level that could damage the chip structure must be avoided. The increase of flow resistance would differ based on the dilution ratio of the blood sample as well. As shown in Equation 1, flow resistance is a function of sample viscosity. According to Fishberg (13), dilution of the blood sample would reduce the blood viscosity significantly. When the dilution is more than five times by volume, the viscosity reduces to a level that is approximate to the viscosity of the diluting solution. When the viscosity decreases, the flow resistance decreases as well, resulting in a decrease in the pressure drop at the same flow rate.

Figure 4C shows the pressure drop at the upstream and downstream capillary when the viscosity was reduced five times compared to whole blood in Figure 4A (in the case of dilution with normal saline, viscosity of blood = 3.5–5.5 cPoise and that of normal saline = 1 cPoise). The results showed that the pressure drop also decreased five-fold at the same flow rate. The mathematic model suggested that the dilution of the blood sample would help prevent an extremely high pressure drop inside the device and enhance its durability during operation.

### 3.2. Optimization of flow rate and blood dilution using a syringe pump

To verify the functionality of the device in a certain range of injecting flow rates, a series of experiments were performed. Pure normal saline was first used to obtain a suitable injecting flow rate. During the experiments, the flow rates were controlled with a syringe pump. The microfluidic chip could withstand the high pressure occurring at all flow rates applied (data not shown). This suggests that the designed chip could be operated at flow rates ranging from 0.5 to 1.8 mL/min without any damage (pressure drop up to 3–4 kPa).

A flow rate of 1 mL/min was chosen for further experiments with suspended blood cells in the diluted sample test. This was mainly due to the ease of operation with a 1 mL syringe that is also suitable for injecting by hand in 1 min to simulate the employment of the device in a field setting.

**Figure 5:**
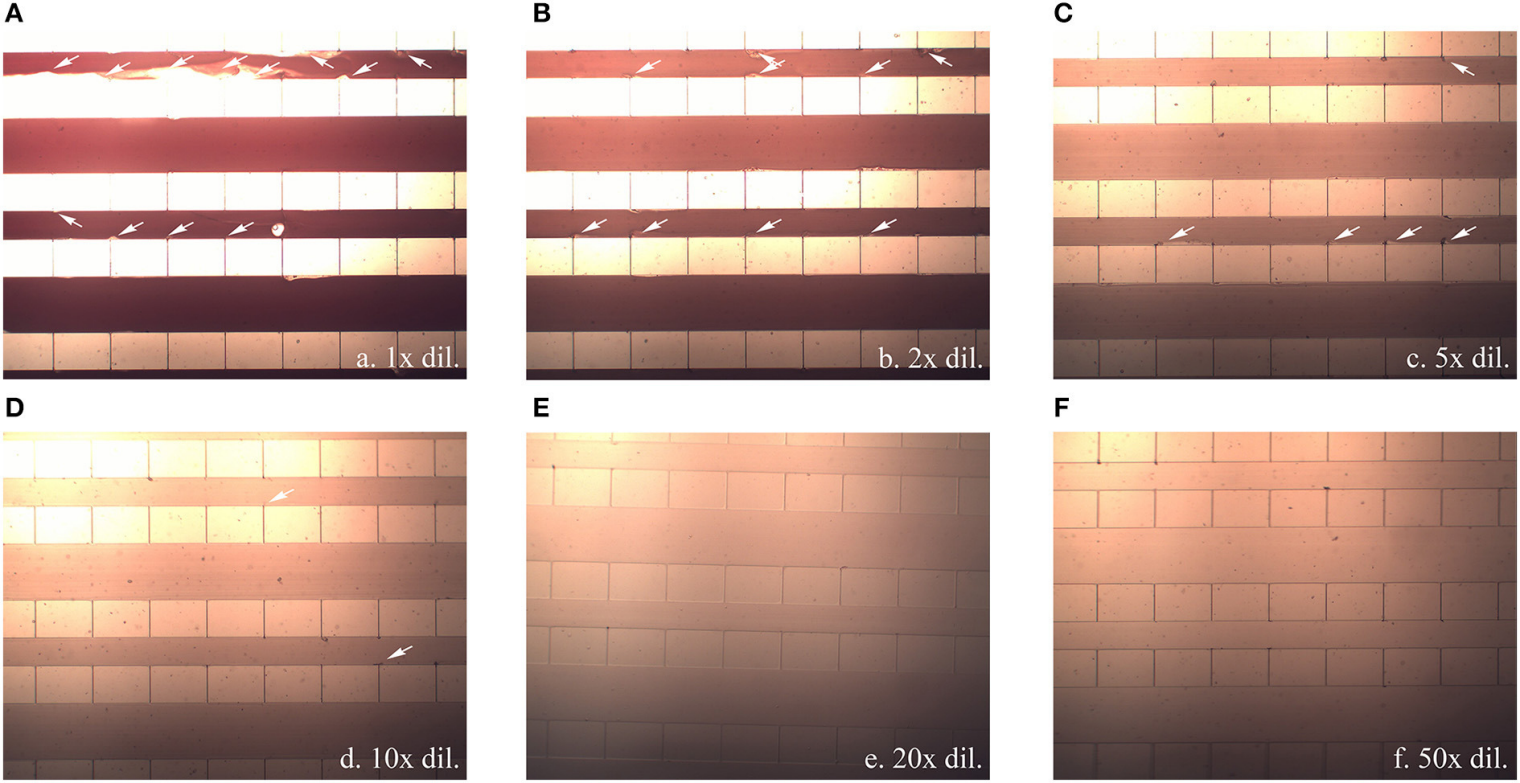
Blood clogging observed inside the flow passage channel (white arrow) when a blood sample diluted at **(A)** 1x, **(B)** 2x, **(C)** 5x, **(D)** 10x, **(E)** 20x, and **(F)** 50x was injected at 1 mL/min. All pictures were captured using 20x magnification.

However, during the experiments with different blood dilution ratios, blood clogging was observed at relatively high blood-cell concentrations (between 1x and 10x) as shown in Figure 5. Not only did severe clogging occur at the capillary openings, fluid leakage due to a crack in the bonding also occurred at dilution ratios of 1x−2x, at which the pressure drop increased to 15–20 kPa. However, clogging gradually decreased as the dilution ratio was increased to 5x−10x, and no clogging was observed at dilution ratios of 20x−100x.

High shear stress occurs when whole blood containing large numbers of blood cells flows through small capillaries, causing blood clogging at the capillary exit. The dilution of the blood sample, leading to the reduction of the sample viscosity and pressure drop as mentioned earlier, would also lower the flow shear stresses across the capillary and reduce the occurrence of cell debris. From the simulation and experimental results, it was suggested that the pressure drop across the capillary should be lower than 3–4 kPa, occurring at an injecting flow rate of 1 mL/min at a dilution higher than 5x, to avoid cell lysis when the blood cells squeezed through the capillary.

In our experiments, the flow rate fluctuated from time to time because of the effort required to push the syringe plunger by hand without controlling the exerted force. Especially in cases where the blood viscosity was high, it was more difficult to control the forces in each attempt. This resulted in fluctuations in the pressure drop as well. Therefore, cell debris and clogging were observed in our experiments even when 5x and 10x dilutions were employed. Beyond that, at blood dilution ratios of 20x−100x, it was easier to control the pushing force since the diluted blood sample was less viscous. In addition to lower viscosity, the flow rate was more uniform, and the possibility of clog formation was reduced. However, with the same amount of blood sample, dilution increases the diagnostic time. Taking these factors into account, a dilution ratio of 20x was chosen for further study.

### 3.3. Detection of microfilariae in the chip

**Figure 6:**
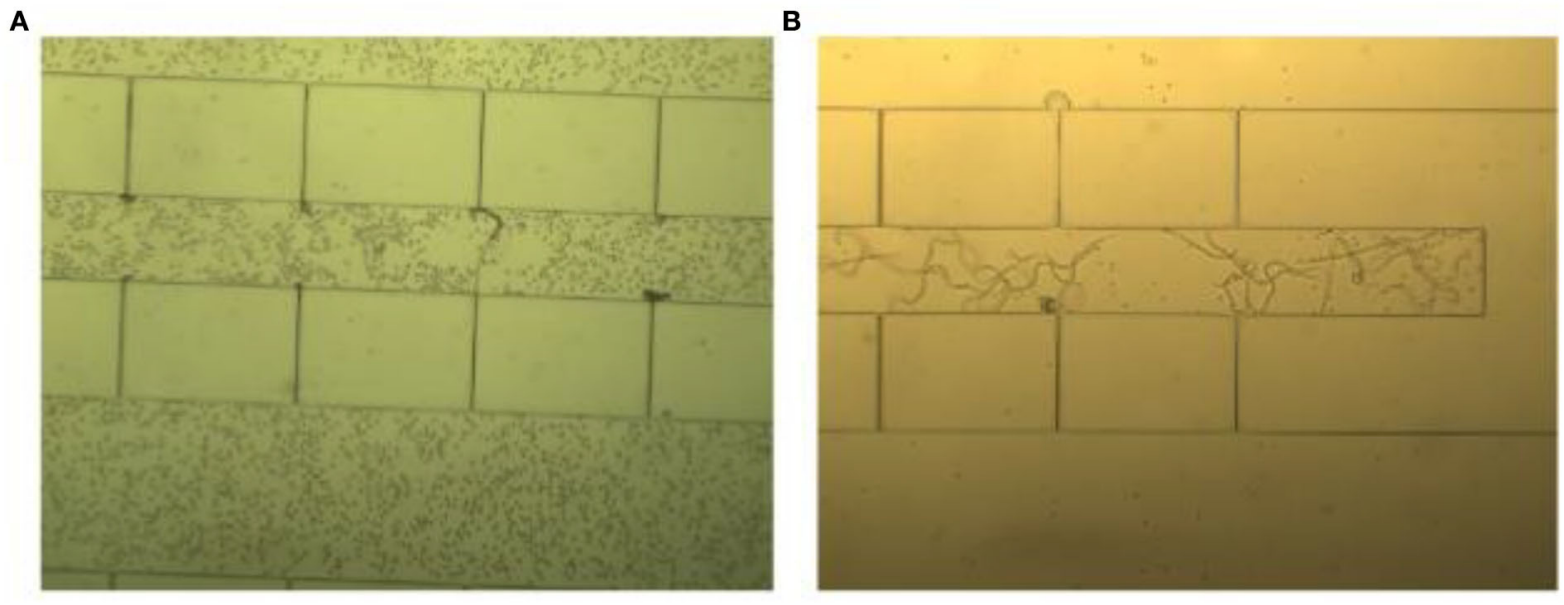
A 20x diluted blood sample was injected by hand in two steps. **(A)** The first step involved blood sample injection, resulting in the retention of blood cells in the passage channel with trapped microfilariae. **(B)** The second step involved washing using normal saline solution, resulting in good visibility of the microfilariae.

Using the chosen blood sample dilution and a 1 mL/min flow rate, 1 mL of the diluted sample was injected by hand within 1 min and the remaining blood cells were washed out using 2 mL normal saline at the same injection rate. In the end, microfilariae were clearly visible in the trapping spot under an inverted light microscope as shown in Figure 6.

The chip showed very high efficiency of microfilaria detection. At dilution ratios of 20x−50x, resulting in an average of 7.5 and 3 microfilariae in 1 mL of the diluted blood sample, the microfluidic chip could still capture and detect their presence as shown in Table 1. These results suggested that this protocol would be suitable for microfilaria detection in low-resource settings such as small hospitals, pet clinics, or farms. After blood sample collection, only a 20x dilution with normal saline is needed before the injection of the diluted sample by hand at a rate of 1 mL/min.

**Table 1:** The number of microfilaria calculated from a blood sample containing 150 Mcf/mL.

**Dilution by volume**	**B:NSS (μl)**	**Number of Mcf (calculated)**	**Number of Mcf in three experiments**			**Number of Mcf (mean value)**
			**Ex. 1**	**Ex. 2**	**Ex. 3**	
1:20	50:950	7.5	4	5	4	4–5
1:50	20:980	3	2	1	2	1–2

After experimentation, the number of microfilariae trapped inside the microfluidic chip was counted. Results are from three replicates (B, blood; NSS, normal saline; Mcf, microfilariae).

### 3.4. Identification of species

**Figure 7:**
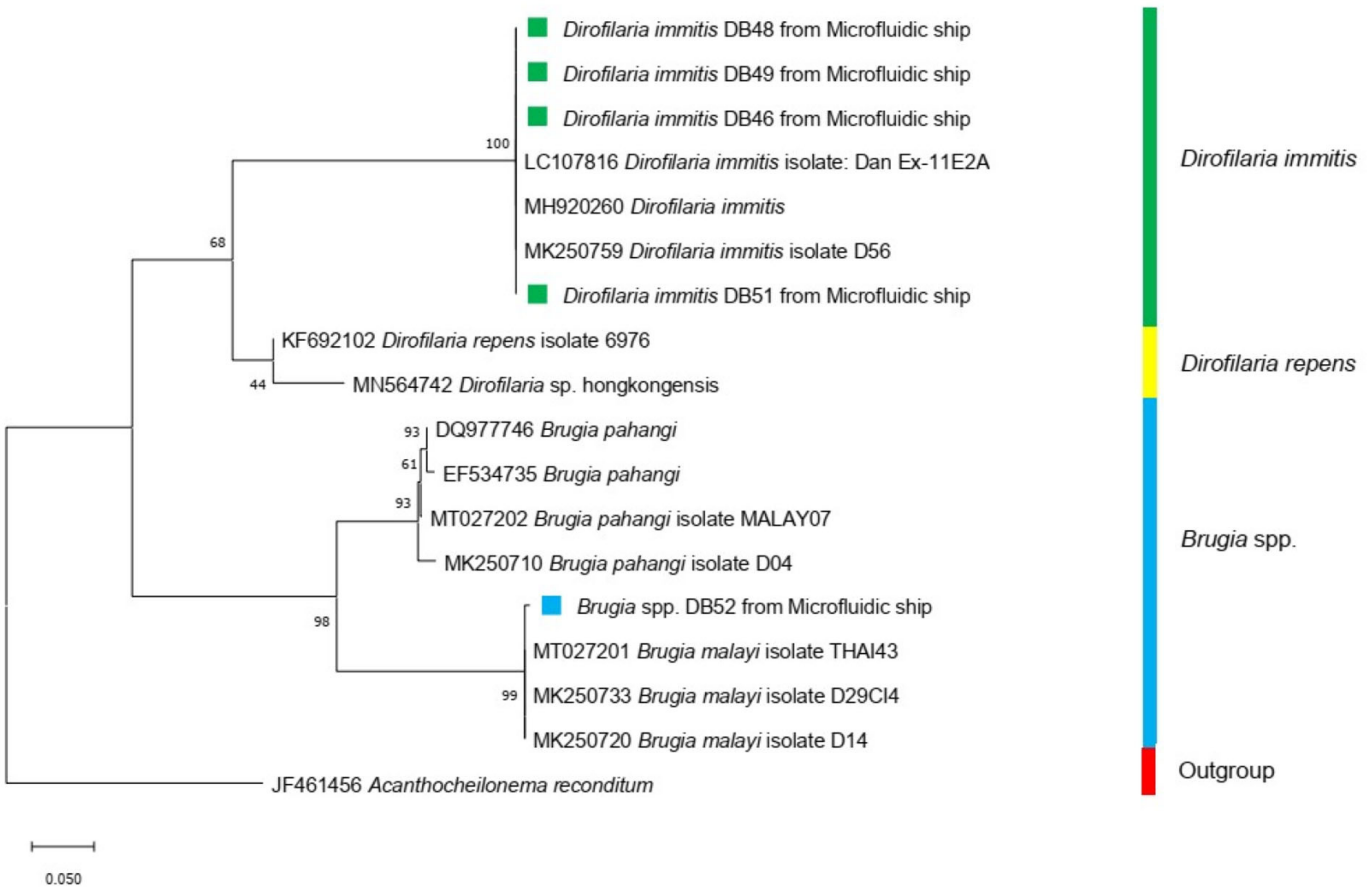
Maximum-likelihood phylogenetic tree using Tamura-Nei for the substitution model disclosed the genetic relationship between *Dirofilaria immitis* (green square) and *Brugia* spp. (blue square) used in this study and other Onchocercidae nematodes based on the COI gene.

After flushing the trapped microfilariae out, species were identified by molecular techniques. We found that the microfilariae of *D. immitis* and *B. pahangi* could be detected by using the rectangular filtering microfluidic chip, and the results of the identified species are shown in Figure 7.

## 4. Discussion

Microfluidic devices have been widely used as diagnostic tools over the past decade due to the efficiency of the identification process and diagnosis, especially for clinical samples (14). Nowadays, point-of-care testing (POCT) is an attractive approach for the diagnosis of diseases near the patient site to save material costs, labor costs, and diagnostic time compared to conventional laboratory-based tests (15). However, simplicity of equipment and operation is required for this purpose.

In other designs, a lytic buffer, syringe pump and a high flow rate are needed to remove other suspended particles. This may cause more microfilariae to squeeze through the filters to the outlet as well, thereby decreasing the device's sensitivity and efficiency (7, 16, 17). The rectangular filtering microfluidic device allowed unwanted particles to pass through at the upstream end, resulting in high sensitivity and efficiency. A high flow rate was unnecessary so that the manual injection of the blood sample into the microfluidic chip would be possible. Given that the nematodes were well isolated, lytic buffer was not needed. The lytic buffer would break red blood cells and white blood cells and produce a lot of cell debris, causing clogging. In the end, without any complicated operational steps, the microfilariae were isolated without harm from chemicals and could simply be flushed out for further *in vitro* culture and drug testing.

In our protocol, only a 20x dilution of the blood sample by volume is needed; the diluted sample is then injected by hand at a rate of 1 mL/min. The blood dilution is an important for reducing blood viscosity and pressure drop inside the chip. Thus, manual injection could be operated without cell lysis or device destruction. Compared to other microfluidic devices, the flow rates of all designs were controlled by the infusion pump. Semi-automated microfluidic device gave good results at flow rate of 6 μl/min for 37 min (7, 17), while flow-through nematode filter (FTNF) biochip showed the best setting value at 0.5 mL/h flow rate for 29 min with pre-treated sample (8). Another platform, Specter 20 (EMD-Millipore) was repurposed to identify microfilariae in human and animals. However, a cost of analysis as POCT should be considered (18).

One drawback of our test protocol is its time-consuming nature when a small number of microfilariae need to be detected. For example, for the detection of 30 microfilariae in a 1-mL blood sample, the examiner may use a 0.1 mL blood sample volume (with an average of three microfilariae) for the 20x dilution, resulting in a 2-mL volume of diluted sample that would take approximately 2 min to inject into the device. However, for the detection of only three microfilariae in 1 mL of blood, with our protocol, approximately 20 min would be required for the diagnosis. This long injection time is impractical by hand, and a syringe pump may be needed in this case. In natural infection of canine heartworm disease, the density of microfilariae in blood was between 17 and 78,417 Mcf/mL (19). However, our result showed the lowest density at 150 Mcf/mL. Therefore, time-consuming may need to be considered when it is applied with very low density of microfilariae in blood sample. To accomplish the device as POCT, many follow-up studies need to be validated such as analytical performance, standardization, repeatability, specificity and sensitivity (20).

The rectangular filtering microfluidic device can be used as a tool for nematodes separation and species identification by RT-PCR. It will be interesting if we can improve the potential of the chip on in-chip identification. Moreover, purified-life microfilariae isolation and concentration are the device's advantages over other conventional methods (21, 22). These might be very impactful for further research.

## 5. Conclusion

We developed a novel method to detect microfilariae that can be performed with high sensitivity in a field setting. The rectangular filtering microfluidic device was fabricated and validated as a POCT for the diagnosis of diseases near the patient site. The protocol was also developed by blood dilution and flow rate optimization. The method can be operated in a short time without any external infusion pump. The microfilariae were efficiently trapped in the microfluidic chip and clearly visible under a light microscope. Therefore, the method is suitable for diagnosis of filariasis in remote area, practical for isolation and identification of microfilariae as well as subsequent culture purposes.

## Data availability statement

The original contributions presented in the study are included in the article/supplementary material, further inquiries can be directed to the corresponding author.

## Ethics statement

The research protocol was approved by the Chulalongkorn University Animal Committee (Approval No. 1731010).

## Author contributions

SA and PP designed the study, conducted the experiments, and wrote the manuscript. AP designed the study and wrote the manuscript. WJe and WS fabricated the microfluidic devices. PT wrote the manuscript. All authors have read and approved the final manuscript.
